# The relation of cytokines TH1, TH2 and TH17 in childhood-onset systemic lupus erythematosus

**DOI:** 10.1186/1546-0096-12-S1-P324

**Published:** 2014-09-17

**Authors:** Karina Peliçari, Mariana Postal, Renata Barbosa, Nailu Sinicato, Fernando Peres, Roberto Marini, Simone Appenzeller

**Affiliations:** 1 Medicine, State University Of Campinas, Campinas, Brazil; 2 Pediatrics, State University Of Campinas, Campinas, Brazil

## Introduction

Childhood-onset Systemic lupus erythematosus (cLES) is an autoimmune disease with a wide spectrum of clinical manifestations, characterized by periods of activity and remission. Hematological and immunological abnormalities are commonly found. Laboratory evaluation, including the cytokine profile, assists in the diagnosis, determination of disease activity and may predict future damage caused by the disease.

## Objectives

To determine the serum levels of Th1 (IL-12), Th2 (IL-6 and IL-10) and Th17 (IL-17) cytokines in cSLE and healthy controls. To evaluate their association whit disease activity, laboratory and treatment features and Mood and anxiety disorders.

## Methods

We included 63 consecutive cSLE patients [median age 18 years (range 11-25)] and 59 healthy controls [median age 20 years (8-33)]. Controls were age and sex-matched to cSLE patients. SLE patients were further assessed for clinical and laboratory SLE manifestations, disease activity [SLE Disease Activity Index (SLEDAI)], damage [Systemic Lupus International Collaborating Clinics/American College of Rheumatology Damage Index (SDI)] and current drug exposures. Total dose of corticosteroids and other immunosuppressant medications used since the onset of disease were calculated by data obtained by careful review of the medical charts.Mood and anxiety disorders were determined through Becks Depression (BDI) and anxiety inventory (BAI). Th1 (IL-12), Th2 (IL-6 and IL-10) and Th17 (IL-17) cytokines levels were measured by ELISA and compared by non-parametric tests.

## Results

Serum IL-6 (p=0.001), IL-10 (p=0.006), IL-12 (p=0.027) and IL-17 (p=0.0001) levels were increased in cSLE patients when compared to healthy controls. IL-6 levels were significantly increased in patients whit active disease (p=0.008). IL-6 (p=0.032) and IL-12 (p=0.028) levels were significantly increased in patients with active nephritis. We observed that IL-17 was associated with migraine (p=0.045), IL-6 with thrombocytopenia (p=0.022) and IL-12 with the presence of anxiety (p=0.048). No association between cytokine levels and SDI scores or medication was observed.

## Conclusion

Cytokines play a central role in cSLE, IL-6 is associated with SLEDAI and may be a biomarker of disease activity, Th1 and Th2 responses may play a role in lupus nephritis and Th1 and Th17 may play a role in neuropsychiatric symptoms in cSLE. Longitudinal studies are necessary to confirm their ability to predict SLE related manifestations.

## Disclosure of interest

K. Peliçari: None declared, M. Postal: None declared, R. Barbosa: None declared, N. Sinicato: None declared, F. Peres: None declared, R. Marini: None declared, S. Appenzeller Grant / Research Support from: FAPESP (2008/02917-0, 2011/03788-2, 2013/09480-5) CNPQ (300447/2009-4 and 471343/2011-0; 302205/2012-8; 473328/2013-5)

